# Survival of *Anisakis simplex* (*s.s.*) L3 exposed to different combinations of acetic acid and sodium chloride: In vitro observations

**DOI:** 10.1016/j.fawpar.2025.e00293

**Published:** 2025-09-27

**Authors:** Kaan Kumas, Cyril Henard, Per Walter Kania, Kurt Buchmann

**Affiliations:** Laboratory of Aquatic Pathobiology, Department of Veterinary and Animal Sciences, Faculty of Health and Medical Sciences, University of Copenhagen, Frederiksberg, Denmark

**Keywords:** Nematoda, Food safety, Zoonosis, Marination

## Abstract

Parasitic nematode larvae within the family Anisakidae are important food-borne parasites with importance for consumer health and international trade. Apart from inactivation of larvae in fish products by heating and/or freezing, marination of herring fillets is widely used. The preferred marination process applies a solution with high contents of sodium chloride and acetic acid. We here describe the direct lethal effect on *Anisakis simplex* (sensu stricto) of different combinations of sodium chloride and acetic acid. We incubated a total of 1440 isolated worm larvae in 36 different combinations of sodium chloride and acetic acid over 8 weeks (w) at 5 °C. Worm mortality was correlated to incubation time, but acetic acid showed the strongest effect when compared to sodium chloride. Full worm mortality was induced by acetic acid (10 %) within 2 w and by 6 % acetic acid after 8 w. Sodium chloride was less effective as worms were alive in 10 % sodium chloride until 4 w and in 8 % until 8 w. A synergistic effect was noted as a combination of 4 % acetic acid and at least 2 % sodium chloride was effective within 6 w. At 8 w 2 % acetic acid combined with 6 % sodium chloride was found lethal for larvae. It cannot be excluded that worm larvae in host tissues will exhibit different mortality rates due to the protective effect of host cells.

## Highlights

• Anisakis simplex L3 sensitivity to acid and salt incubation described.
• Acetic acid has the strongest effect on nematode larvae.
• Sodium chloride has effect but to a lower extent.
• Lethal combinations of acetic acid and sodium chloride are depicted.

## 1 Introduction

Parasitic nematodes within the family Anisakidae are listed among the 24 most important food-borne parasites with high international trade impact (Levsen and Berland, 2012; WHO/FAO, 2014; Bouwknegt et al., 2018; Bao et al., 2019; FDA, 2022; Cipriani et al., 2024). The family comprises the genera *Anisakis, Phocanema* (syn. *Pseudoterranova*) and *Contracaecum*, representing parasitic nematodes with complex life cycles (Buchmann and Mehrdana, 2016; EFSA, 2024a). Marine mammals host the adult stage and invertebrates and fish serve as first and second paratenic hosts, respectively, carrying the third infective larval stage (L3) (Cipriani et al., 2022). The zoonotic importance of these larval parasites relates to their potential to infect human consumers following ingestion of raw or undercooked seafood products (Strøm et al., 2015; Baptista-Fernandes et al., 2017; Suzuki et al., 2021; Nordholm et al., 2020; EFSA, 2024a). The disease caused by them (without specifying the genus of the pathogen) are commonly termed anisakidosis, whereas infection with a representative for the genus *Anisakis* is termed anisakiosis or anisakiasis (Buchmann and Mehrdana, 2016). In addition to their impact on human health, parasites also cause marketability problems and thereby influence the fishing industry negatively (Llarena-Reino et al., 2015; Bao et al., 2019). A recent analysis disclosed that no fishing grounds can be considered free from zoonotic parasites (EFSA, 2024b), which emphasizes the need for preventive measures when handling wild caught fish for human consumption. Therefore, food authorities have placed focus on prevention by reinforcing regulations, such as EU-regulation 853(2004), requiring seafood products to be inspected and treated in order to inactivate or kill parasites (EFSA, 2024a). One of the genera, *Anisakis,* comprises a range of marine nematode species such as *A. simplex, A. pegreffi, A. typica, A. physeteris, A. berlandi, A. paggiae, A. nascetti and A. brevispicula* (Mattiucci et al., 1997; Mattiucci et al., 2014). Various species representatives of the genus can be present in a wide range of marine fish species from the Arctic to Antarctic (Klimpel et al., 2010: Mattiucci et al., 2018; Severin et al., 2020; Bao et al., 2025). The parasites are natural parts of the ecosystems, which is reflected by the fact that *Anisakis simplex* has a complex life cycle including crustaceans as first paratenic host, fish and cephalopods as second paratenic host (Køie et al., 1995), whereas cetaceans (Cipriani et al., 2022) and pinnipeds (Kumas et al., 2025a) serve as definitive hosts. Three species within the *Anisakis* genus, *A. simplex, A. typica* and *A. pegreffi*, have a confirmed zoonotic potential, as they may infect humans following consumption of raw or undercooked *Anisakis* infected seafood products (Buchmann and Mehrdana, 2016; Shamsi and Barton, 2023; EFSA, 2024a). The human disease results from worm penetration of the ventricular or intestinal mucosa, in some cases with extra-intestinal migration, with resulting inflammatory local and/or systemic reactions (Suzuki et al., 2021). The first confirmed case of anisakiasis in Europe occurred in the Netherlands in the 1950s, where live *A. simplex* larvae were recovered from human patients following their consumption of lightly salted Atlantic herring *Clupea harengus* (Van Thiel et al., 1960). In the individual fish the parasitic nematode larvae are usually located in body cavity and organs, but some migrate further and invade the fish musculature (Levsen and Berland, 2012; Cipriani et al., 2024; Kumas et al., 2024a; Bao et al., 2025). Apart from heat inactivation (60 °C, 1 min, as applied in baking, cooking, frying and hot smoking) a range of methods have been applied to inactivate the larvae in the fish product intended to be eaten in a raw or semi-raw condition (EFSA, 2024a). These include freeze storage (−20 °C, 24 h) and marination. The latter method includes storage over several weeks in a mixture of various ingredients including different types of acid, salt, spices and oils (EFSA, 2024a). Standard procedures for inactivation of *A. simplex* in Atlantic herring apply incubation of fillets in brines with sodium chloride and acetic acid as developed by Karl et al. (1995). These authors based their recommendations on experimental incubation of herring fillets containing larvae. However, the effective concentrations reported were high for both sodium chloride and acetic acid and the direct effect on the worm larvae (without fish musculature) is less well known. Other studies have elucidated killing of isolated nematode larvae in different salt concentrations (Sánchez-Monsalvez et al., 2005; Oh et al., 2014; Šimat and Trumbić, 2019). However, species identification was not always performed by the needed molecular techniques as requested (EFSA, 2024a). As it cannot be excluded that different species within the genus *Anisakis* react differently to salt and acid we find it necessary to identify the *Anisakis* species (by both morphological and molecular methods) when performing survival tests. In addition, the chemical composition (e.g. lipid content) of the fish incubated in the brine may affect sensitivity of the larvae (Karl et al., 1995). We have therefore for the present study identified the exposed nematodes by use of morphological and molecular methods. Then a systematic evaluation was performed elucidating the survival over 8 weeks (w) of isolated *A. simplex* sensu stricto (*s.s.*) third-stage larvae exposed to 36 combinations of sodium chloride and acetic acid at 5 °C. The lethal salt-acid combinations for these zoonotic larvae (in isolation) at different timepoints 2–8 w post-incubation may be used as a supporting tool in the industry.

## 2 Materials and methods

### 2.1 Experimental design

The overall aim was to evaluate the survivability of *Anisakis simplex* exposed to various combinations of acetic acid and sodium chloride solutions. The experiment was conducted in quadruplicate at 5 °C, utilizing 36 different solutions that included various combinations of acetic acid and sodium chloride. For each combination 40 (4 × 10) worm larvae were placed in 50 mL Falcon tubes containing 40 mL acetic acid/sodium chloride solution (each tube with 10 larvae). A tube with 10 worms was sampled from each combination at four different time-points (2, 4, 6 and 8 w). In case of complete mortality (all 10 worms in a tube found) at the inspection time point, the remaining worms in the additional tubes with the same acid/salt combination were also examined to ensure comprehensive data collection.

### 2.2 Nematode recovery

A batch of Atlantic herring *Clupea harengus*, captured in the North Sea (ICES A4) by a commercial trawler, was transported under cooled conditions to the university laboratory (Frederiksberg, Copenhagen area). The fish were dissected and live *A. simplex* (L3) isolated from the body cavity and transferred to Petri dishes with tap water at room temperature (20 °C) until they had left their encapsulation of host cells. Activity of the worms was recorded according to Kumas et al. (2024b) and only motile nematodes without encapsulation were selected. Thus, a total of 1440 larvae were selected to be used in the experiment. These were randomly distributed into 144 tubes as described above. Due to their high viability and lack of encapsulation by host tissue these larvae may not be fully representative of larvae protected by tissues in fish fillets. However, they may contribute with baseline information on the sensitivity of clean *A. simplex* larvae to marinade components. Additional motile larvae were preserved in 96 % ethanol (KiiltoClean A/S, Denmark) and kept at 4 °C until morphological and molecular identification.

### 2.3 Evaluation of viability

The study was based on 1440 larvae distributed into 144 tubes. Four of these (4 × 10 larvae) were used to test each combination of acetic acid and sodium chloride. At each sampling time point, a Falcon tube with 10 nematode larvae from a particular acid/salt combination was examined by placing worms in labeled Petri dishes containing tap water (1 h at 21 °C and then 1 h at 37 °C), a procedure which activates most live worms (Kumas et al., 2024b). At each temperature active nematodes were noted, then removed from the group and preserved in 96 % ethanol. For a final test of viability of the nematodes, if they showed no movements at 37 °C, they were placed in glass beakers (staining cups, volume 4 mL) containing pre-heated glycerin-gelatine (Buchmann, 2007) at 56 °C and observed under the dissection microscope (magnification X40). If the larvae showed any activity they were considered alive, but if no movements were seen they were recorded as dead. If all 10 larvae in the first tube examined at a specific time point were recorded as dead, the remaining 3 tubes were examined correspondingly. Mortality of 100 % (0 % survival) was only recorded if all larvae in all tubes were dead.

### 2.4 Morphological and molecular identification

A subsample of 8 specimens was randomly selected from the batch of isolated motile larvae (L3) and placed in 96 % ethanol. Each individual worm was aseptically divided into three parts (anterior, middle and posterior). Genus level identification was performed by morphological analyses. The anterior and posterior parts were cleared with lactic acid (Merck, Denmark) and mounted with glycerin-gelatine on a microscope slides (SuperFrost® Plus, VWR International A/S, Denmark) and assigned to genus level. Molecular analyses were performed from the middle part of the nematodes, which were incubated in lysis buffer with Proteinase K. PCR was conducted targeting mitochondrial DNA (mtDNA) and subsequently sequenced. DNA was purified by using QIAamp DNA mini kit (Qiagen, Denmark) according to the manufacturer's instructions with a modification (using elution buffer 50 μL instead of 200 μL). PCR was conducted by using BioRad T100 Thermal Cycler (Bio-Rad Laboratories, Denmark). The reaction volume of each PCR product was 60 μL composed of 28.6 μL of RNase-free water (cat.no. 12060346, Fisher Scientific, Denmark), 6 μL of forward primer, 6 μL of reverse primers (Tag Copenhagen, Frederiksberg, Denmark) (both 10 mM), 6 μL of 10 mM dNTPmix (Applied Biosystems™ GeneAmp™ dNTP Blend (100 mM) Fisher Scientific, Slangerup, Denmark), 6 μL of 10× Reaction buffer, 1.8 μL of 50 mM MgCl2, 0.6 μL of DNA Polymerase (all three BIOTAQ DNA Poly-merase, cat.no BIO-21060, Saveen & Werner ApS, Jyllinge, Denmark) and 5 μL of the sample. The mitochondrial gene *cox2* was targeted according to Nadler and Hudspeth (2000) by using forward primer 211F (5′-TTTTCTAAGTTATATAGATTGRTTTYAT-3′) and reverse primer 210R (5′-283 CACCAACTCTTAAAATTATC-3′). PCR conditions were 1) pre-denaturation at 95 °C for 5 min, 2) amplification cycles included denaturation at 95 °C for 30 s with a touch down procedure using a sequence of 2 cycles at 53 °C, 52 °C, 51 °C, 50 °C, 3 cycles at 49 °C, 48 °C, 47 °C and 35 cycles at 46 °C for annealing with each step for 45 s. PCR products were visualized by 2 % agarose gel electrophoresis and purified by using Illustra™ GFX™PCR DNA and Gel Band Purification Kit (VWR International A/S, Denmark) according to the manufacturer's instructions. The CLC-Main Workbench v20.0.4 (QIAGEN, Denmark) software was used to perform sequence analyses.

### 2.5 Statistics

Evaluation of nematode survival and comparison between groups at each time point was performed by the Kruskal-Wallis test with Dunn's multiple comparison. Differences between effects of sodium chloride concentrations at different acetic acid concentrations were assessed along effect differences between acid concentrations at different salt concentrations. The larval survival in the 0 % acetic acid and 0 % sodium chloride group served as controls. A general probability level of 5 % was applied (*P* *<* *0.05*). All statistical analyses were performed by using Graph Pad Prism 10. 5. 0 (www.graphpad.com, USA).

## 3 Results

### 3.1 Identification of parasites

All larvae were identified to genus level *Anisakis* by their morphological characteristics (larval tooth, excretory pore anterior to nerve ring, absence of intestinal caecum, mucron in posterior end and ventriculus without appendage). All subsamples were identified *A. simplex* sensu stricto *(s.s.*) by molecular methods targeting the mitochondrial gene *cox2.* We obtained eight PCR products, all 630 bp long (including primer binding sites), which were submitted to GenBank and obtained accession nos. PV975770 → PV975777. BLAST search at NCBI showed that the sequences showed highest identity towards *A. simplex s.s.* and the identities ranged from 99.36 % to 99.84 % (Table 1).

**Table 1:** Details on the molecular identification of *Anisakis simplex* L3 used in the study. PCR was performed targeting mtDNA. Identities of the partial *cox2* sequences of this study towards GenBank entries. For each specimen only one of the entries exhibiting the highest identity is shown.

Parasite of this study			Identity analysis	
Worm id	Species	GenBank acc.no.	Percentage	GenBank acc.no.
365_01	*A. simplex* s.s	PV975770	99.50	OQ448184
365_02	*A. simplex* s.s	PV975771	99.84	PP203000
365_03	*A. simplex* s.s	PV975772	99.52	PQ126420
365_04	*A. simplex* s.s	PV975773	99.50	MN961158
365_05	*A. simplex* s.s	PV975774	99.52	PQ126423
365_06	*A. simplex* s.s	PV975775	99.68	PP202994
365_07	*A. simplex* s.s	PV975776	99.52	PV200021
365_08	*A. simplex* s.s	PV975777	99.36	MT989559

**Fig. 1:**
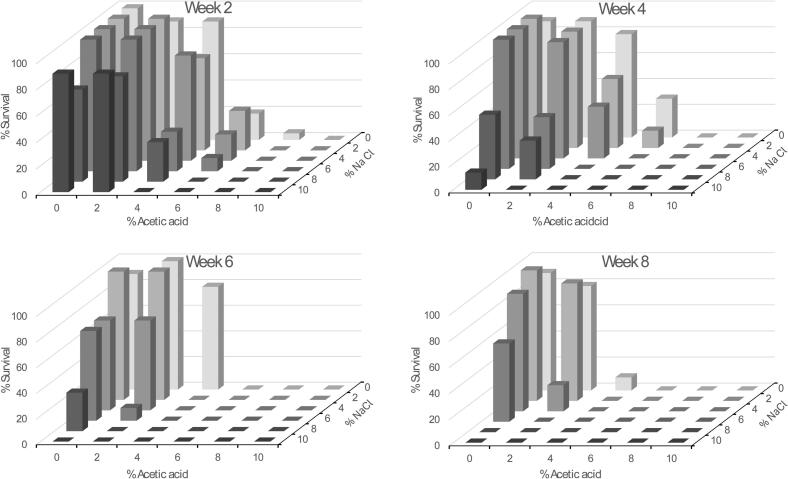
3D column diagram showing survival of *Anisakis simplex* L3 over 2, 4, 6 and 8 w when exposed in vitro to different combinations of acetic acid and sodium chloride concentrations (5 °C).

**Table 2:** *Anisakis simplex* L3 kept in different combinations of acetic acid and sodium chloride concentrations. Test of NaCl concentration against 0 % NaCl. Comparison of nematode survival over weeks (2, 4, 6, 8) Between different NaCl concentrations under different acetic acid concentrations. Numbers indicate significant difference, and ns indicates no significant difference between groups (Control group as %0 NaCl) compared (Kruskal-Wallis with Dunn's multiple comparisons test, *P* *< 0.05*).

	NaCl	Acid 0 %	Acid 2 %	Acid 4 %	Acid 6 %	Acid 8 %	Acetic acid 10 %
Week 2	0 % vs. 2 %	ns	ns	ns	ns	ns	ns
	0 % vs. 4 %	ns	ns	ns	ns	ns	ns
	0 % vs. 6 %	ns	ns	0.0233	ns	ns	ns
	0 % vs. 8 %	0.0383	ns	0.0233	ns	ns	ns
	0 % vs. 10 %	ns	ns	<0.0001	ns	ns	ns
Week 4	0 % vs. 2 %	ns	ns	ns	ns		
	0 % vs. 4 %	ns	ns	ns	0.0006		
	0 % vs. 6 %	ns	ns	<0.0001	0.0006		
	0 % vs. 8 %	ns	0.0350	<0.0001			
	0 % vs. 10 %	0.0001	<0.0001				
Week 6	0 % vs. 2 %	ns	ns	<0.0001	ns		
	0 % vs. 4 %	ns	ns	<0.0001			
	0 % vs. 6 %	ns	<0.0001				
	0 % vs. 8 %	0.0240	<0.0001				
	0 % vs. 10 %	0.0069					
Week 8	0 % vs. 2 %	ns	ns				
	0 % vs. 4 %	ns	0.0215				
	0 % vs. 6 %	ns	ns				
	0 % vs. 8 %	<0.0001					
	0 % vs. 10 %

**Table 3:** *Anisakis simplex* L3 kept in different combinations of acetic acid and sodium chloride concentrations. Test of acetic acid concentration against 0 % acetic acid. Comparison of nematode survival over weeks (2, 4, 6, 8) Between different acetic acid concentrations under different NaCl concentrations. Numbers indicate significant difference, and ns indicates no significant difference between groups (Control group as 0 % acetic acid) compared (Kruskal-Wallis with Dunn's multiple comparisons test, *P* *< 0.05*).

	Acetic acid	NaCl 0 %	NaCl 2 %	NaCl 4 %	NaCl 6 %	NaCl 8 %	NaCl 10 %
Week 2	0 vs. 2	ns	ns	ns	ns	ns	ns
	0 vs. 4	ns	ns	ns	0.0005	0.0304	<0.0001
	0 vs. 6	0.0003	0.0016	0.0002	<0.0001	<0.0001	<0.0001
	0 vs. 8	<0.0001	<0.0001	<0.0001	<0.0001	<0.0001	<0.0001
	0 vs. 10	<0.0001	<0.0001	<0.0001	<0.0001	<0.0001	<0.0001
Week 4	0 vs. 2	ns	ns	ns	0.0014	ns	ns
	0 vs. 4	ns	0.0332	0.0024	<0.0001	0.0004	
	0 vs. 6	0.0040	<0.0001	<0.0001	<0.0001		
	0 vs. 8	ns					
	0 vs. 10						
Week 6	0 vs. 2	ns	ns	ns	0.0017	0.0296	
	0 vs. 4	ns	<0.0001	0.0024			
	0 vs. 6	<0.0001	0.0025				
	0 vs. 8						
	0 vs. 10						
Week 8	0 vs. 2	ns	ns	0.0055	ns		
	0 vs. 4	0.0007					
	0 vs. 6						
	0 vs. 8						
	0 vs. 10						

### 3.2 Nematode survival

Nematode survival (following incubation in various combinations of acetic acid and sodium chloride) was assessed every two weeks over a period of 8 w (Fig. 1, Supplementary file S1). The 0 % acetic acid/0 % sodium chloride combination groups served as controls for the main groups (Table 2, Table 3). At 10 % acetic acid, all nematodes were dead by week 2, and therefore no significant differences were seen between groups regardless of salt concentrations. At 6 % and 8 % acetic acid, significant differences were observed across all sodium chloride concentrations except 6 % acid with 2 % salt. When the acetic acid concentration reached 4 %, worm mortality in all salt concentrations were significantly different from control. At 2 % acetic acid, significant differences were observed when the salt concentrations were ≥ 6 %. At 0 % acetic acid, significantly elevated worm mortalities were observed when the sodium chloride concentrations were ≥ 8 %. At the lowest salt concentrations (0 % and 2 %), there was no significant difference in the 2 % acetic acid concentration compared to the control group (0 % acid). However, in these salt concentrations the acid concentration of 4 % and higher affected nematode survival significantly. In salt concentrations at 4 % and higher, all acid concentrations affected survival significantly (Fig. 1, Table 2, Table 3, Supplementary file S2).

### 3.3 Survival rates by time of exposure

Nematode survival was examined at 2, 4, 6 and 8 w. Acid concentration was the primary lethal factor and the salt concentration the secondary factor (Fig. 1, Supplementary file S1). Acid at 10 %, 8 % and 6 % killed all larvae at week 2, week 4 and week 6, respectively. In a salt concentration of 10 % and 8 % it took 6 and 8 w, respectively, to inactivate all larvae (Supplementary file S2). By 8 w, nematodes exposed to 0 % acid were still alive if they were under 6 % salt concentration. Similarly at 2 % acid nematodes were still alive under 4 % salt concentrations and at 4 % acid concentrations only few nematodes were still alive in one group where there was no salt concentration. Following incubation in 6, 8 and 10 % acid all nematodes were found dead. In lower acid concentrations (4, 2 and 0 %) addition of salt (2 %, 6 % and 8 %, respectively) was needed to reach 100 % worm mortality (Fig. 1, Supplementary file S2).

## 4 Discussion

*Anisakis simplex* s.s. is one of the prominent species within the nematode family Anisakidae. It is known to be prevalent in the Atlantic herring in which a part of the worm infrapopulation resides in the musculature (Levsen and Berland, 2012; Kumas et al., 2024a). According to the EU-regulation 853/2004 these nematode larvae must be inactivated before they reach the consumers (EFSA, 2024a), which is due to the zoonotic potential of anisakid nematode larvae in fish products (Buchmann and Mehrdana, 2016). One of the inactivation methods is the use of a marination process, which is an incubation of the product within a solution of sodium chloride and acetic acid. As a basis for developing precise and safe marination methods we need information on the sensitivity of the *A. simplex* larvae to different combinations of salt and acid. This knowledge may guide the industry when developing brines for the industrial storage of potentially infected fish products. It cannot be excluded that different species within the genus *Anisakis* respond differently to acid and salt exposure. Therefore, we found it necessary to perform a precise identification to species level by use of molecular identification techniques before survival investigations were performed. We aimed at providing a baseline for the susceptibility/sensitivity of worm larvae to marinade components. Therefore, the present study was based on isolated nematode larvae, which were rinsed from host tissue, cells and debris at room temperature before incubation. This may on the other hand be a limitation of the study. Worm larvae in fish products are often deeply embedded in organs and encapsulated by several layers of host cells (Kumas et al., 2025b), and even the chemical composition of the fish organ may influence the sensitivity of worms to acid and salt in marinades. Thus, Karl et al. (1995) reported that survival rates of *Anisakis* sp. larvae were affected by the fat content of the fish material tested, and that an elevated lipid content of the products increased worm survival. Further, this type of exposure experiment may further benefit from a blinded test design, which may be applied in future studies. The present study described that live and highly motile larvae became inactivated within 2–8 w depending on the concentrations used. This type of data can be applied in future development of suitable marination protocols, which may affect the taste and marketability less than previously adopted procedures. Thus, currently the industry relies mainly on the study by Karl et al. (1995), who described the survival of *Anisaki*s sp. larvae in herring fillets using various methods. Tests were performed with focus on a common German procedure and traditional Danish procedure as well as some additional brine solutions. In the German procedure, herrings were stored at 3 °C in a brine consisting of 14 % salt, 7 % acetic acid and < 0.1 % H_2_O_2_, a process which killed *Anisakis* larvae over 35 d. In the traditional Danish procedure, herrings were kept in a 10 % salt solution at 12 °C for 17 h. The fillets were then mixed with a brine containing 5 % acetic acid and 10 % salt and stored at 3 °C, a procedure, which killed nematodes in 42 d. Additional work by Karl et al. (1995) showed that *Anisakis* sp. larvae were dead after 4 w in a solution containing 6.3 % salt and 3.7 % acetic acid. When *Anisakis* larvae were tested following storage in 2 % acetic acid and 4–5 % salt larvae died after 17 w. If the salt concentration was raised to 6–7 % the worms were inactivated within 10–12 w and in 8–9 % salt they died after 5–6 w.

The present study demonstrated that *A. simplex s.s.* larvae were inactivated after 14 days of exposure to a solution containing 8 % acetic acid and 10 % salt. Similarly, they were dead after 14 days in both 4 % and 6 % acetic acid combined with 10 % salt. When exposed to 4 % acetic acid and 6 % salt, larvae died within 28 d (4 w). Under conditions of 2 % acetic acid combined with 4 % sodium chloride, larvae were alive at 8 w. However, when 2 % acetic acid was combined with either 6 % or 8 % sodium chloride, the larvae died within 8 w. Sánchez-Monsalvez et al. (2005) reported that *Anisakis* larvae (species not specified) in anchovies were dead after 13 d when stored in a brine solution containing 6 % acetic acid and 12 % salt, and in a brine solution with 10 % acetic acid and 12 % salt, the larvae died after 5 d. The data indicated a lethal effect of acid and salt on worm survival but they cannot be directly compared to our results due to another experimental design. Similarly, Arcangeli et al. (1996) demonstrated that *Anisakis* larvae in sardines marinated with 6 % acetic acid and 10 % sodium chloride (stored at 4 °C) were dead after 13 d, and those data corresponds partly to our results.

Fish products spoil quickly in nature due to their chemical composition. Therefore, methods such as freezing, canning, drying, smoking, marination and salting have been applied to preserve fish products (Ghaly et al., 2010; Sampels, 2015). Marination of seafood products was even described in the 7th century (Shenderyuk and Bykowski, 1991). However, too high concentrations of the ingredients (acid, salt) may affect the taste adversely. In addition, a long storage time before the product reaches the retail level, and thereby consumers, may challenge profitability in the processing industry. Therefore, a finer adjustment of brines, based on and assisted by the presented data, may be applicable in the future.

## 5 Conclusions

Data for worm survival in different combinations of acetic acid and sodium chloride are useful when designing brines. The present study lists combinations of acetic acid and sodium chloride concentrations which are lethal for *A. simplex* L3 within 2, 4, 6 and 8 w. However, the limitation of the data presented in the present work is the use of isolated larvae free from protective host material. As host tissue may offer worms some protection against acid and salt, future corresponding studies could benefit from a combination of the present approach with exposure of parasites in situ (in fish tissues such as musculature). It should be further noted that the method used for evaluation of worm viability is important for reaching reliable and comparable results in survival tests. Several approaches to assess viability have been applied (EFSA, 2024a). The main point in this study is that lack of worm motility (even with mechanical stimulation) at room temperature (20 °C) or even human body temperature (37 °C) was not a reliable measure for viability. Thus, some of the non-motile worm larvae, which did not show movements at these lower temperatures (20 °C and 37 °C) could be activated when transferred to glycerine-gelatine at 56 °C. We therefore recommend this additional viability test to be considered in future viability studies.

The following are the supplementary data related to this article.

Supplementary File S1 Heatmaps showing differential survival (at 2, 4, 6 and 8 w) of *Anisakis simplex* L3 exposed to different combinations of acetic acid and sodium chloride concentrations. Supplementary File S1

Supplementary File S2 Tables showing survival of *Anisakis simplex* L3 as percentage of live nematodes exposed to acetic acid-NaCl solutions at week 2–4 (A, B, C, D). Numbers below the percentage indicate live and dead / nematodes respectively. Not available (NA) indicates all the nematodes were already eliminated earlier weeks. Supplementary File S2

## Declaration of generative AI in scientific writing

The authors declare that no Generative AI was used in the creation of this manuscript.

## CRediT authorship contribution statement

**Kaan Kumas:** Writing – review & editing, Writing – original draft, Visualization, Methodology, Investigation, Formal analysis, Data curation. **Cyril Henard:** Writing – review & editing, Writing – original draft, Methodology, Investigation. **Per Walter Kania:** Writing – review & editing, Writing – original draft, Visualization, Investigation, Formal analysis, Data curation. **Kurt Buchmann:** Writing – review & editing, Writing – original draft, Validation, Supervision, Resources, Project administration, Methodology, Investigation, Funding acquisition, Formal analysis, Conceptualization.

## Funding

The present study was supported by the 10.13039/100008396Danish Ministry of Food, Agriculture and Fisheries under the program GUDP with grant no. 34009–22-2100 (OPTIKVAL).

## Declaration of competing interest

The authors declare that the research was conducted in the absence of any commercial or financial relationships that could be construed as a potential conflict of interest.
