# Supplementary figures and images for: Survival of Anisakis simplex (s.s.) L3 exposed to different combinations of acetic acid and sodium chloride: In vitro observations
Source: Food Waterborne Parasitol. 2025 Sep 27;41:e00293. doi: 10.1016/j.fawpar.2025.e00293 (PMC12517068; doi:10.1016/j.fawpar.2025.e00293)

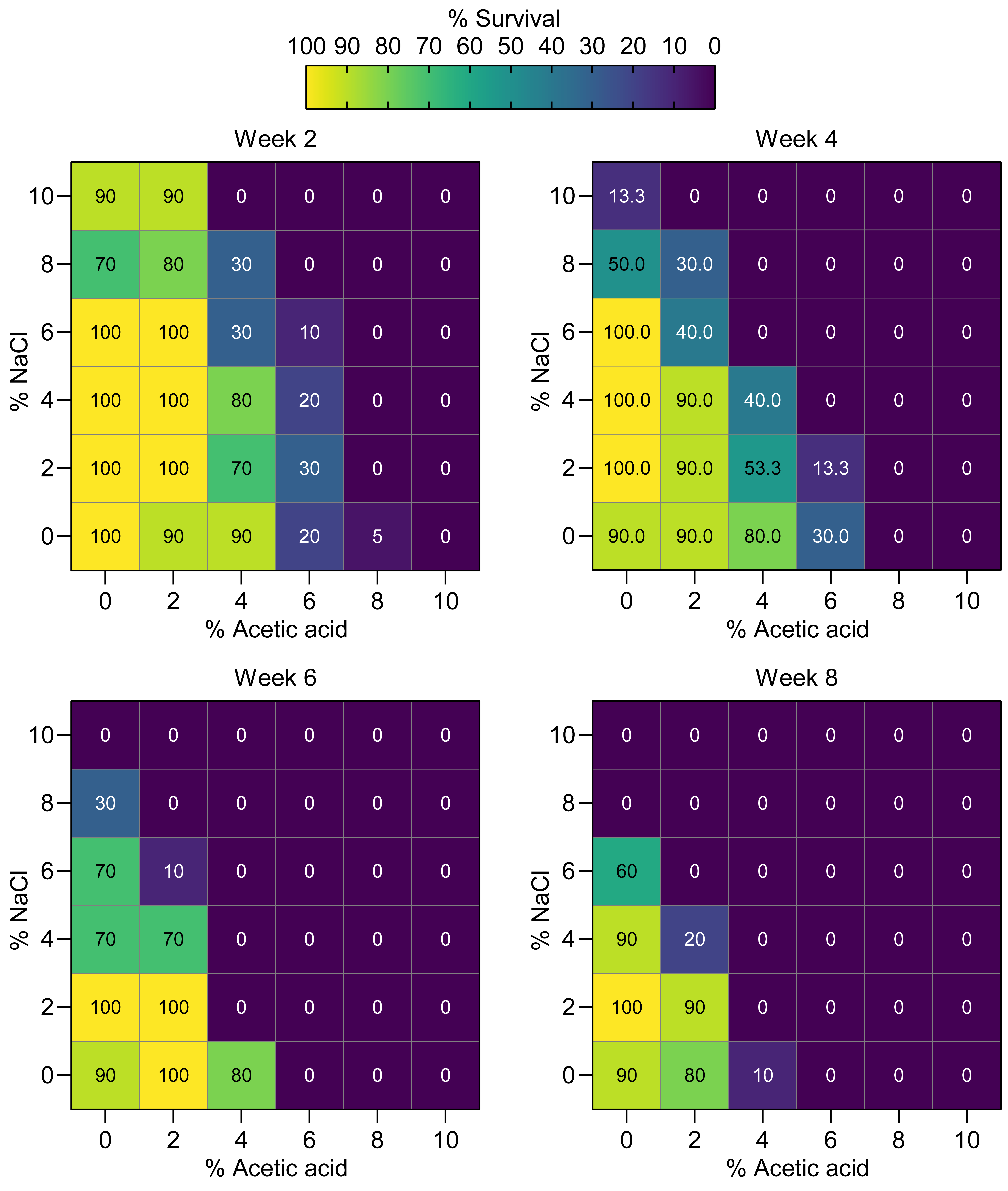

Supplement: Supplementary File S1 — Heatmaps showing differential survival (at 2, 4, 6 and 8 w) of Anisakis simplex L3 exposed to different combinations of acetic acid and sodium chloride concentrations. [file mmc1.zip › Suppl. S1. Survival - Heat Maps.tif]
