# Supplementary material for: Survival of Anisakis simplex (s.s.) L3 exposed to different combinations of acetic acid and sodium chloride: In vitro observations
Source: Food Waterborne Parasitol. 2025 Sep 27;41:e00293. doi: 10.1016/j.fawpar.2025.e00293 (PMC12517068; doi:10.1016/j.fawpar.2025.e00293)
Supplement: Supplementary File S2 — Tables showing survival of Anisakis simplex L3 as percentage of live nematodes exposed to acetic acid-NaCl solutions at week 2–4 (A, B, C, D). Numbers below the percentage indicate live and dead / nematodes respectively. Not available (NA) indicates all the nematodes were already eliminated earlier weeks. [file mmc2.docx]

**Supplementary file S2 – Table A.** Percentage of live nematodes exposed to acetic acid-NaCl solutions at week 2. Numbers below the percentage indicate live and dead / nematodes respectively. Not available (NA) indicates all the nematodes were already eliminated earlier weeks.

|  | Acetic acid 0% | Acetic acid 2% | Acetic acid 4% | Acetic acid 6% | Acetic acid 8% | Acetic acid 10% |
| --- | --- | --- | --- | --- | --- | --- |
| NaCl 0% | 100% (10/0) | 90% (9/1) | 90% (9/1) | 20% (2/8) | 5% (2/38) | 0% (0/40) |
| NaCl 2% | 100% (10/0) | 100% (10/0) | 70% (7/3) | 30% (3/7) | 0% (0/40) | 0% (0/40) |
| NaCl 4% | 100% (10/0) | 100% (10/0) | 80% (8/2) | 20% (2/8) | 0% (0/40) | 0% (0/40) |
| NaCl 6% | 100% (10/0) | 100% (10/0) | 30% (3/7) | 10% (1/9) | 0% (0/40) | 0% (0/40) |
| NaCl 8% | 70% (7/3) | 80% (8/2) | 30% (3/7) | 0% (0/40) | 0% (0/40) | 0% (0/40) |
| NaCl 10% | 90%  (9/1) | 90% (9/1) | 0% (0/40) | 0% (0/40) | 0% (0/40) | 0% (0/40) |

**Supplementary file S2** **– Table B.** Percentage of live nematodes exposed to acetic acid-NaCl solutions at week 4. Numbers below the percentage indicate live / dead nematodes, respectively. Not available (NA) indicates all the nematodes were already eliminated earlier weeks.

|  | Acetic acid 0% | Acetic acid 2% | Acetic acid 4% | Acetic acid 6% | Acetic acid 8% | Acetic acid 10% |
| --- | --- | --- | --- | --- | --- | --- |
| NaCl 0% | 90% (9/1) | 90% (9/1) | 80% (8/2) | 30% (9/21) | 0% (0/2) | NA |
| NaCl 2% | 100% (10/0) | 90% (9/0) | 53.3% (16/14) | 13.3% (4/26) | NA | NA |
| NaCl 4% | 100% (10/0) | 90%  (9/1) | 60% (18/12) | 0% (0/30) | NA | NA |
| NaCl 6% | 100% (10/0) | 40%  (4/6) | 0% (0/30) | 0% (0/30) | NA | NA |
| NaCl 8% | 50% (5/5) | 30% (3/7) | 0% (0/30) | NA | NA | NA |
| NaCl 10% | 13.3% (4/26) | 0% (0/30) | NA | NA | NA | NA |

**Supplementary file S2 – Table C.** Percentage of live nematodes exposed to acetic acid-NaCl solutions at week 6. Numbers below the percentage indicate live / dead nematodes, respectively. Not available (NA) indicates all the nematodes were already eliminated earlier weeks.

|  | Acetic acid 0% | Acetic acid 2% | Acetic acid 4% | Acetic acid 6% | Acetic acid 8% | Acetic acid 10% |
| --- | --- | --- | --- | --- | --- | --- |
| NaCl 0% | 90% (9/1) | 100% (10/0) | 80% (8/2) | 0% (0/9) | NA | NA |
| NaCl 2% | 100% (10/0) | 100% (10/0) | 0% (0/16) | 0% (0/4) | NA | NA |
| NaCl 4% | 70%  (7/3) | 70%  (7/3) | 0% (0/12) | NA | NA | NA |
| NaCl 6% | 70%  (7/3) | 10%  (2/18) | NA | NA | NA | NA |
| NaCl 8% | 30% (3/7) | 0% (0/20) | NA | NA | NA | NA |
| NaCl 10% | 0% (0/4) | NA | NA | NA | NA | NA |

**Supplementary file S2** **– Table D.** Percentage of live nematodes exposed to acetic acid-NaCl solutions at week 8. Numbers below the percentage indicate live / dead nematodes, respectively. Not available (NA) indicates all the nematodes were already eliminated earlier weeks.

|  | Acetic acid 0% | Acetic acid 2% | Acetic acid 4% | Acetic acid 6% | Acetic acid 8% | Acetic acid 10% |
| --- | --- | --- | --- | --- | --- | --- |
| NaCl 0% | 90% (9/1) | 80% (8/2) | 10% (1/9) | NA | NA | NA |
| NaCl 2% | 100% (10/0) | 90% (9/1) | NA | NA | NA | NA |
| NaCl 4% | 90%  (9/1) | 20%  (2/8) | NA | NA | NA | NA |
| NaCl 6% | 60%  (6/4) | 0%  (0/2) | NA | NA | NA | NA |
| NaCl 8% | 0% (0/10) | NA | NA | NA | NA | NA |
| NaCl 10% | NA | NA | NA | NA | NA | NA |
